# Supplementary material for: Single Nucleotide Polymorphisms in the Bovine TLR2 Extracellular Domain Contribute to Breed and Species-Specific Innate Immune Functionality
Source: Front Immunol. 2021 Dec 23;12:764390. doi: 10.3389/fimmu.2021.764390 (PMC8732954; doi:10.3389/fimmu.2021.764390)
Supplement: Supplementary file 1 [file DataSheet_1.pdf]

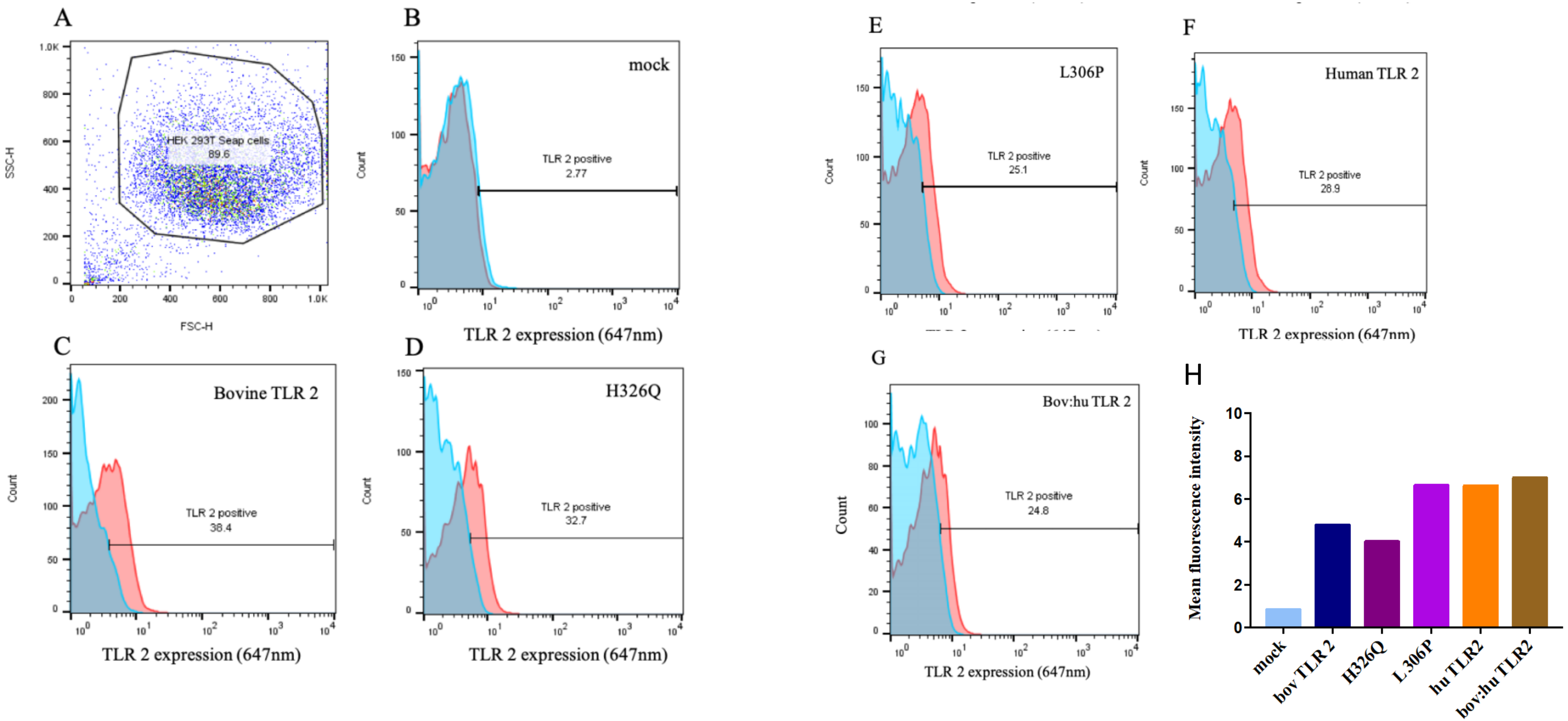

Figure S1: TLR2 receptor expression in HEK 293 SEAP cells upon transient transfection. Upon transient transfection with different TLR2 constructs, surface expression of TLR2 by HEK 293 SEAP cells was detected by immune-labelling using human anti-bovine CD282 antibody or mouse anti-human CD282 antibody conjugated with Alexa Fluor 647. HuCAL Fab-dHLX-MH antibody was used as a negative control.. A total of 10,000 events were recorded per construct by a FACS Calibur E3160 using Cell Quest Pro acquisition software (BD Biosciences, UK). Data was analysed using FlowJo V10 (FlowJo LLC, USA). Gating of SEAP cell population was based on forward-side scatter (A) and gating strategy was used for all constructs (B-G). Images represent the sum of three independent transient transfections per construct. The mean fluorescent intensity of cells from three independent transient transfections per construct is shown (H)

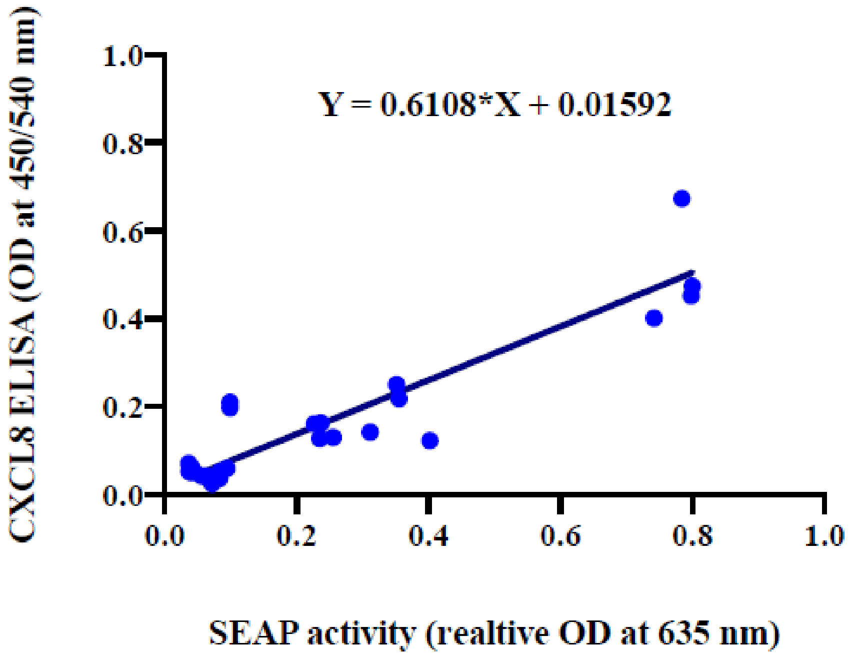

Supplementary Figure S2: Correlation of ODs measured in SEAP reporter and CXCL8 ELISA assay

ODs measured in the same supernatants of transfectants stimulated with FSL-1 at 100 ng/ml (Invivogen, USA), Pam^3CSK^4 at 1 mg/ml (Invivogen, USA) and their controls, measured in the SEAP assay and human CXCL8 ELISA assay using Quantikine Human CXCL8/IL-8 ELISA kit (R&D Systems, USA) were analysed for correlation between both assays. Spearman's correlation was implemented and found to be significant with a strong degree of correlation (r=0.7537, p<0.0001). To display this correlation, a dot plot fitted with Deming's regression line was drawn. Deming's regression line and equation are shown for one representative experiment. Data analysed and displayed with GraphPad Prism V8 (GraphPad Inc., USA)

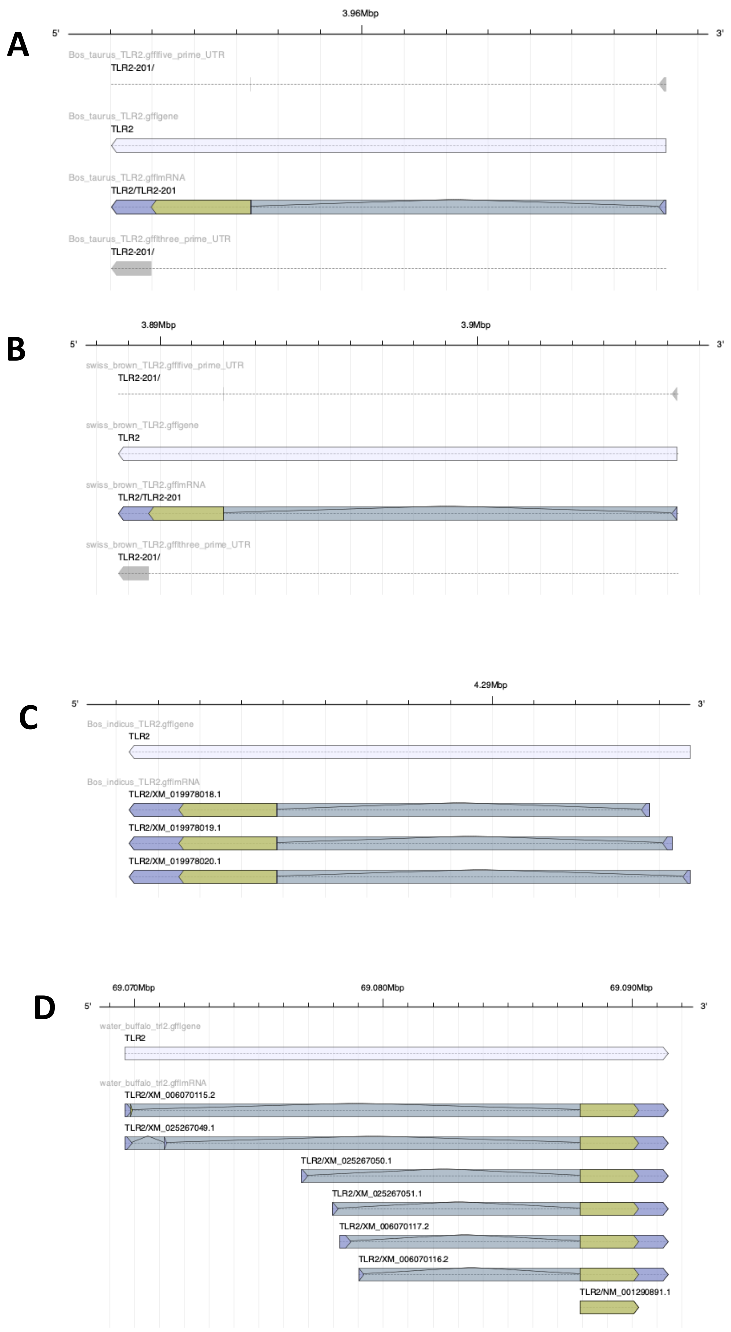

Supplementary Figure S3: TLR2 Gene Models

Gene models were created for TLR2 to represent *B. taurus* **(A)**, Brown Swiss **(B)**, *B. indicus* **(C)** and *B. bubalis* **(D)** identify the number of possible transcripts.

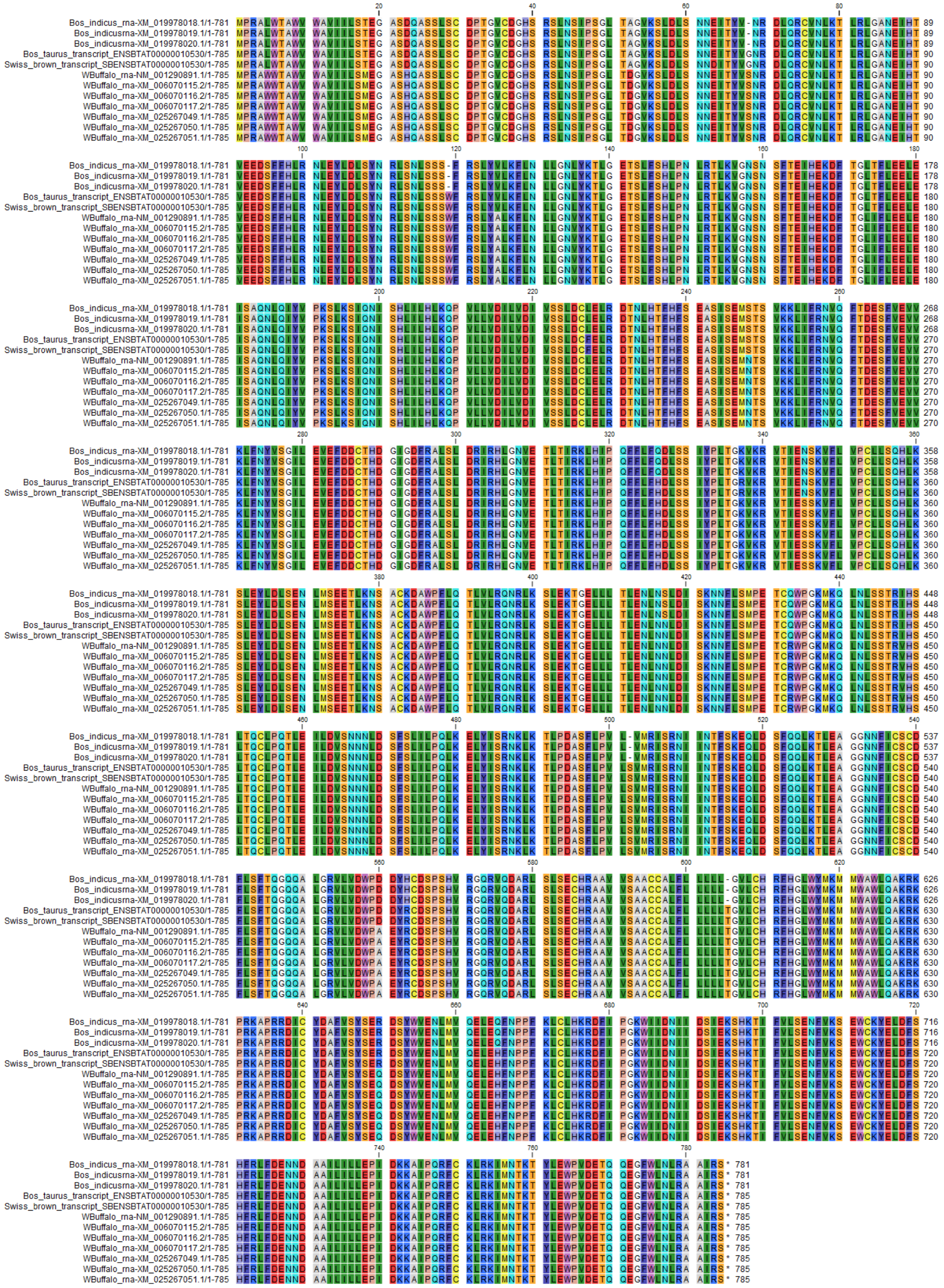

Supplementary Figure S4: TLR2 CDS Alignment

Coding sequences for TLR2 for each transcript defined in TLR2 gene models were extracted and aligned with ClustalW and translated to protein sequences.

Notes:

Sites where Brown Swiss differs from all others.
63 (D>E) GAG > GAT transversion
D=ASP Aspartic acid
E=GLU Glutamic acid

Sites where Brown Swiss and Bos taurus differ from all others

Sites where Brown Swiss and Bos taurus differ from Bos indicus Site (Bos taurus/Bos indicus)
211 (I>V)
227 (F>L)
337 (R>K)

Sites where Bos indicus differs from all others (B. indicus aa first)
119 (->W)
326 (Q>H)
417 (S>N)
502 (->S)
563 (H>R)
605 (->T)
665 (Q>H)

Variable sites
68 (-/G/S)
Bos indicus –
Bos taurus G
Brown Swiss G
Water Buffalo S
